# Endosonography-Guided Pancreatic Duct Drainage for Chronic Pancreatitis: A Case Report and Review

**DOI:** 10.1155/2010/517864

**Published:** 2010-05-27

**Authors:** Kei Ito, Naotaka Fujita, Yutaka Noda, Go Kobayashi, Takashi Obana, Jun Horaguchi, Shinsuke Koshita, Yoshihide Kanno

**Affiliations:** Department of Gastroenterology, Sendai City Medical Center, Sendai, Miyagi 983-0824, Japan

## Abstract

A 50-year-old man was admitted to our department, complaining of epigastric pain and high fever. CT revealed a pseudocyst at the pancreatic head with upstream dilatation of the pancreatic duct (PD) and fluid collection surrounding the pancreas. Endosonography-guided PD drainage (ESPD) was performed because of unsuccessful ERCP. With a curved linear array echoendoscope, a 7.2 F catheter was placed in the PD. Laboratory data showed improvement in a few days and revealed disappearance of the fluid collection. Ten days after ESPD, a 7 F stent was placed in the PD via the puncture tract across the papilla of Vater followed by transpapillary replacement with a 10 F stent. CT showed a reduction in diameter of the PD and disappearance of the pseudocyst. ESPD is a feasible and useful procedure in selected patients with chronic pancreatitis showing stenosis of the main PD when transpapillary approach is impossible.

## 1. Introduction

 The cause of pain in patients with chronic pancreatitis (CP) is multifactorial, one of which is regarded to be pancreatic ductal hypertension. Ductal decompression is advocated for patients with pain and a markedly dilated duct. Endoscopic approach to CP is gaining wider application [1, 2]. Transpapillary pancreatic duct (PD) stenting has been reported to be useful for relieving pain in such patients [3]. However, in some patients transpapillary approach is impossible because of severe inflammation involving the duodenum, tight PD stricture, or altered anatomy. 

 Endosonography- (ES-) guided pancreatography for a diagnostic purpose in a patient with failed transpapillary approach was first reported by Harada et al. in 1995 [4]. Several reports on the application of this technique from diagnostic purpose to therapeutic one, such as ES-guided pancreatic duct drainage (ESPD), have been published [5–12]. We applied ESPD in patients with CP and the results were quite encouraging. Herein, its details are reported with a review of the literature.

## 2. Case Report

 A 50-year-old man was admitted to our department, complaining of epigastric pain and high fever. A diagnosis of alcohol-induced CP was made 10 years earlier. Laboratory data on admission showed the following abnormalities: serum amylase: 2909 IU/L (normal range: 42–130 IU/L); protronbin time: 34% (70–100%); Fibrinogen: <50 mg/dl (150–400); DD-dimer: 24.11 *μ*g/ml (0.00-1.00 *μ*g/ml). Abdominal enhanced computed tomography (CT) revealed a pseudocyst 2.5 cm in diameter at the pancreatic head with upstream dilatation of the PD and fluid collection surrounding the pancreas. 

**Figure 1:**
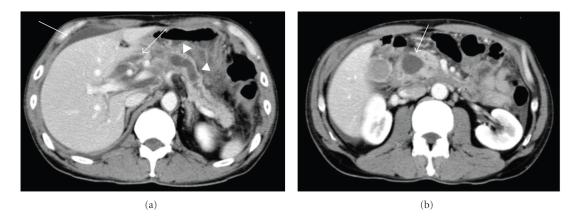
Enhanced CT image. (a) The dilated main pancreatic duct (PD) (arrowhead) and fluid collection (arrow) surrounding the pancreas and the liver were demonstrated. (b) A pseudocyst (arrow) at the pancreatic head was revealed.

**Figure 2:**
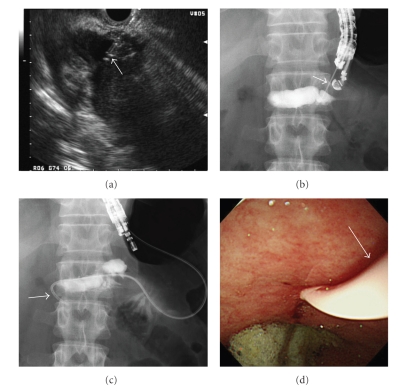
Endosonography-guided pancreatic duct drainage (ESPD): (a) endosonographic image, (b, c) X-ray image, and (d) endoscopic image. The PD was punctured via the body of the stomach with a 19-gauge needle (arrow) (a, b). A 7.2 F nasopancreatic duct drainage catheter (arrow) was placed (c, d).

 Based on the diagnosis of acute pancreatitis, he received drip infusion and pharmacological therapy, including administration of antibiotics (cefoperazone–sulbactam 2 g/day). However, laboratory data did not improve and enhanced CT revealed increase in fluid collection surrounding not only the pancreas but also the liver (Figure 1). Surgical intervention was considered, but the risk was estimated too high by a consultant surgeon. Therefore endoscopic retrograde cholangiopancreatography (ERCP) with PD stenting was attempted; however, scope insertion into the second portion of the duodenum was impossible because of severe inflammation in the duodenum. After obtaining a written informed consent, ESPD was attempted. Following visualization of the dilated PD with a curved linear array echoendoscope (GF-UC240P: Olympus Optical Co., Ltd. Tokyo, Japan), the PD was punctured via the body of the stomach with a 19-gauge needle (Echotip: Cook Corp., Winston-Salem, NC) (Figures 2(a) and 2(b)). After removal of the core needle and aspiration of serous pancreatic juice, contrast agent was injected via the sheath catheter in order to obtain pancreatography for the guide of PD stenting. Pancreatography revealed a PD stenosis at the pancreatic head. A 0.035-inch guidewire (Jagwire: Boston Scientific Corp., Natick, MA, USA) was introduced into the sheath catheter and inserted into the PD. Subsequent to removing the sheath catheter while leaving the guidewire in situ, the puncture tract was dilated with a balloon catheter 4 mm in diameter (Maxforce: Boston Scientific), and a 7.2 F nasopancreatic duct drainage (NPD) catheter (HANACO MEDICAL Co., Ltd, Saitama, Japan) was placed (Figures 2(c) and 2(d)). The procedure time was 36 minutes. The patient developed no symptoms related to the procedure and his initial complaints soon disappeared. Laboratory data showed improvement and revealed that fluid collection disappeared on enhanced CT in a few days. 

**Figure 3:**
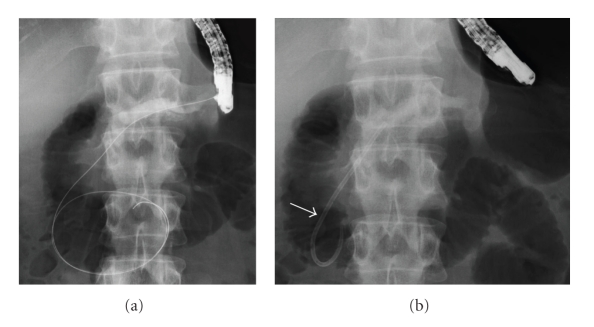
Prograde PD stenting: (a, b) X-ray image. Ten days after ESPD, a guidewire was passed through the papilla of Vater (a). A 7 F stent with a single duodenal pigtail (arrow) was inserted into the PD via the puncture tract (b).

**Figure 4:**
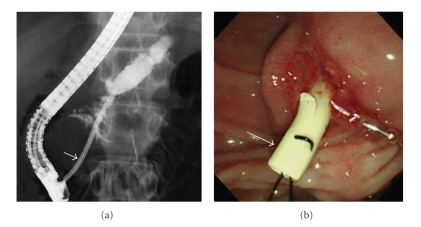
(a) X-ray image, (b) endoscopic image. The stent was transpapillarily replaced with a 10 F straight stent (arrow) one week later.

**Figure 5:**
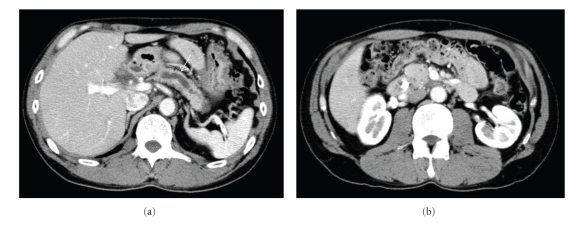
Enhanced CT image. A reduction in diameter of the PD (arrow) and the disappearance of the pseudocyst were demonstrated.

 Ten days after ESPD expecting mature fistula formation, a 0.035-inch guidewire (Jagwire: Boston Scientific Corp., Natick, MA, USA) was passed via the NPD catheter across the papilla of Vater using a duodenoscope (TJF-240: Olympus) under fluoroscopic guidance (Figure 3(a)). A 7 F stent with a single duodenal pigtail (Cathex, Co., Ltd., Tokyo, Japan) was inserted into the PD via the puncture tract after dilating the PD stenosis in the pancreatic head with a 7 F tapered catheter (Figure 3(b)). The puncture tract spontaneously closed without any intervention. The stent was transpapillarily replaced with a 10 F straight stent (Cathex, Co., Ltd.) one week later (Figure 4). Cytologic specimens obtained from the stricture of the PD were negative for malignancy. Six months later after the stent exchange, enhanced CT showed a reduction in diameter of the PD (from 13 to 9 mm) and the pseudocyst disappeared (Figure 5). The stent was then removed. The patient remained asymptomatic during a follow-up of 24 months. Pancreatic function (both endocrine and exocrine) was not evaluated.

## 3. Discussion

 Endoscopic treatment can provide short-term relief of abdominal pain in selected patients with CP. Surgical drainage has been considered to be more invasive than endoscopic therapy and has a high morbidity and mortality. Cahen et al. [13] recently conducted a prospective randomized controlled trial, which elucidated that surgical drainage of the pancreatic duct was more effective than endoscopic treatment in patients with PD obstruction due to CP. Complication rate was similar in the two treatment groups. However, surgical treatment is not indicated in patients with a severely ill condition as shown in this particular case, and endoscopic treatment is recommended for such patients. Although transpapillary approach is desirable for endoscopic treatment of CP, it is not always possible, especially in patients with inflammatory stenosis of the duodenum due to CP or with a history of Whipple's procedure.

 Linear array echoendoscopes provide high-quality images of the pancreas and adjacent organs and allow therapeutic applications such as ES-guided celiac blockade [14], pancreatic pseudocyst drainage [15], and biliary drainage [16]. Harada et al. [4] first reported ES-guided pancreatography in patients who had undergone pylorus-preserving pancreatoduodenectomy for chronic pancreatitis. Several authors reported ES-guided drainage of the PD in patients with inaccessible PD (Table 1) [5–12]. ESPD allows several types of applications such as pancreatogastrostomy (PG)/pancreatobulbostomy (PB), rendezvous procedure, and temporary utilization. A limitation of the rendezvous procedure is that it can be attempted only in patients in whom the papilla of Vater is accessible by endoscopy. Moreover, scope exchange or repositioning is necessary after guidewire insertion into the duodenum via the puncture tract through the papilla of Vater. Therefore, this complicated procedure implies a risk of guidewire migration during scope change and requires additional time. Furthermore, there is also a risk of pancreatic juice leakage. Fujita et al. [17] reported temporary utilization of ES-guide biliary drainage, for gaining access to the bile duct in order to deploy a self-expandable metal stent via the transhepatic route. We also inserted a PD stent through the papilla of Vater ten days after ESPD. To avoid leakage of pancreatic juice, temporary NPD catheter was placed for ten days for the creation of a stable sinus tract. After maturation of the sinus tract, a 7 F stent was inserted into the PD via the puncture tract across the papilla of Vater. Although EUS-related procedures have a risk of complications such as leakage, bleeding, and infection, this new technique, “antegrade PD stenting”, is feasible and useful for selected patients with unsuccessful ERCP. When guidewire insertion through the stricture is unsuccessful, PG/PB are recommended as a salvage procedure.

 ES-guided PG/PB has been reported by several authors [6, 8, 9]. Tessier et al. [8] reported the effectiveness and safety of PG/PB in 36 patients with inaccessible PD. Technical success rate and clinical response rate were 92% and 69% of the patients, respectively. These procedures are reported to be feasible and effective in the management of pain in patients with a dilated MPD in whom the transpapillary approach is not feasible. However, the information on long-term outcome after PG/PB remains unknown.

 ES-guided procedures have a low potential risk of bleeding as color Doppler evaluation is utilized for determining the route of puncture. Reported ESPD-related complication rate was relatively low (0–15%). However, these techniques should be limited for tertiary centers with trained endoscopists, backed up by experts in interventional radiology and skilled surgeons. Moreover, endoscopists who perform ES-guided procedures should be knowledgeable of complications such as leakage, bleeding, and infection after the procedure. If ES-guided procedures are impossible to perform, percutaneous drainage of the PD with a US/CT guidance, which is thought to have a high morbidity, may be a salvage procedure.

 In conclusion, ESPD-related procedures are feasible and useful in selected patients in whom conventional ERCP or surgical treatment is impossible. Further investigations including indications and long-term outcome of these procedures are awaited. Further development of accessory devices specialized for this technique is also necessary.

## Figures and Tables

**Table 1:** Reports of endosonography-guided pancreatic duct drainage.

Author (year)	*N*	Patients	Methods	Initial stent	Technicl success rate	Complication		Clinical response rate
						Early	Late	
Bataille (2002)	1	Chronic pancreatitis	Rendezvous	7 F	100% (1)	No	—	100% (1)
François (2002)	4	Chronic pancreatitis Pancreatic divisum PD rupture	PG	6 F	100% (4)	No	50% (2) SM	75% (3)
Mallery (2004)	4	Recurrent pancreatitis Pancreatic fistula Pancreatic divisum	Rendezvous	7 F	25% (1)	No	—	—
Tessier (2007)	36	Chronic pancreatitis Surgical diversion PD rupture	PG (26) PB (7)	6-7 F	92% (33)	14%(5) 2, Sever*	55% (20)SD	69% (25)
Kahaleh (2007)	13	Chronic pancreatitis Gallstone pancreatitis Surgical diversion	PG (10)	7 F	77% (10)	15% (2)†	No	77% (10)
S$\overset{ˇ}{a}$ftoiu (2007)	1	Chronic pancreatitis	Rendezvous	7 F	100% (1)	No	No	100% (1)
Keenan (2007)	1	Ampullary adenoma (Papillectomy)	Rendezvous	5 F	100% (1)	No	—	100% (1)
Gleeson (2007)	1	Chronic pancreatitis	Rendezvous	7 F	100% (1)	No	No	100% (1)

*N* number; PD: pancreatic duct; PG: pancreatogastrostomy; PB: pancreatobulbostomy; SM; stent migration; SD: stent dysfunction; *1: hematoma; 1: acute pancreatitis; †1: bleeding; 1: perforation.
